# Cheminformatics-Based Anticoagulant Study of Traditionally Used Medicinal Plants

**DOI:** 10.18869/acadpub.ibj.21.6.400

**Published:** 2017-11

**Authors:** Mahdi Alikhani Pour, Soroush Sardari, Ali Eslamifar, Abid Azhar, Mohammad Rezvani, Milad Nazari

**Affiliations:** 1 Drug Design and Bioinformatics Unit, Department of Medical Biotechnology, Biotechnology Research Center, Pasteur Institute of Iran, Tehran 13164, Iran; 2 Department of Clinical Research, Pasteur Institute of Iran, Tehran 13164, Iran; 3 Dr. A. Q. Khan Institute of Biotechnology and Genetic Engineering (KIBGE), University of Karachi, Karachi 75270, Pakistan; 4 Department of Agricultural Biotechnology, Payame Noor University, Tehran, Iran

**Keywords:** Cheminformatics, Cardiovascular diseases, Medicinal plants

## Abstract

**Background:**

Medicinal plants, as a complementary medicine, have been used to treat various diseases since ancient times. These plants have numerous beneficial applications and are the source of certain conventional drugs. In diseases such as stroke and ischemia, which are caused by several factors, abnormal coagulation is an important causative factor. Accordingly, novel and effective therapies such as herbal remedies should be practiced to prevent such lethal diseases.

**Methods:**

Using the available databases such as Google Scholar and PubMed, the previously reported anticoagulant compounds and plants possessing anticoagulant activity were identified and collected in two separate lists. Next, the fast and cost-effective cheminformatics methods incorporated in PubChem were applied to detect some compounds similar to reported anticoagulants. Subsequently, 15 native medical plants of Iran containing the potential anticoagulants were selected. The selected plants were purchased and chopped, and the potential compounds were extracted by ethanol. Then three concentrations of extracts (1, 10, and 100 µg per ml) were made. Finally, anticoagulant effect of the selected plants was evaluated by *in vitro* prothrombin time and activated partial thromboplastin time coagulation tests.

**Results:**

Among the 15 selected medicinal plants, three plants, including *Terminalia bellirica* (*P*=0.0019), *Astragalus arbusculinus* (*P*=0.0021), and *Origanum vulgare* (*P*=0.0014) showed a more promising anticoagulant effect in comparison to the control.

**Conclusion:**

The anticoagulant activity was identified for the first time in these three plants. Further *in vivo* study and mechanism of action assay are required to be performed on these three plants, which could be suitable candidates for use as natural anticoagulant medicines.

## INTRODUCTION

Plants are a great source of ingredients with numerous beneficial uses, including therapeutic and pharmaceutical applications, which are in use since ancient times. Although they are known safe and show many therapeutic effects in a vast range of illnesses, they may have harmful effects due to a great amount of compounds, which some are unknown and some are toxic[1]. Nevertheless, medicinal plants are the only available option to treat diseases in some regions[2]. The modern drugs have more usages than herbal medicines in the community, but in most cases, they have more adverse side effects, especially those made from chemical materials; for example, bleeding complications in warfarin use[3]. Therefore, the use of medicinal plants may be a better choice due to negligible harmful effects[4]. It should be noted that those who use their medicine along with medicinal plants should be cautious due to the increased effect of the medicine. There are several reports regarding the harms of concurrent use of medicinal plants and modern drugs[5,6], and it should be taken seriously.

Heart attack and stroke are the most important causes of death worldwide. In individuals with high blood pressure, hyperlipidemia, and relevant disorders, there is an increased risk of heart attack and stroke, which have irrecoverable consequences. The blood coagulation system is a necessary process to stop excessive bleeding and to prevent hemostasis imbalance[7,8]. Despite the high importance of the normal coagulation, any incorrect blood clot, especially in vital organs such as brain, heart, and liver, will cause serious injuries, and in acute incidences, it will lead to death. For instance, the number of deaths caused by heart attack and strokes has increased in Iran recently[9]. Hence, finding safe and effective therapeutic approaches to prevent and treat such diseases, particularly with the use of medicinal plants, which are safer, cheaper, and more available, is an important goal that we are seeking to achieve with the help of novel and fast cheminformatics methods[10] in the present study.

## MATERIALS AND METHODS

### Gathering information regarding anticoagulant compounds and plants

To gather a list of all the known anticoagulant compounds and plants, databases such as Google Scholar and PubMed were searched with appropriate keywords, which are listed in our previous work[11]. Accordingly, we prepared two separate lists from anticoagulant compounds and plants reported previously[11].

### Cheminformatics step: finding similar compounds and substances

To find similar compounds to the reported anticoagulant compounds, similarity search was carried out according to the name or the chemical structure of the identified anticoagulant compounds in the PubChem structure search engine. The PubChem similarity search uses Tanimoto calculation and the PubChem constructed binary fingerprint to discover related structures according to the specified threshold of similarity. In this respect, the specified threshold of similarity was set at 90%[12]. Afterward, the similar compounds found by PubChem were used to select suitable medicinal plants to evaluate their anticoagulant effect.

### Selecting, purchasing, and extracting the candidate plants

The selection of candidate plants was based on their similarity in having one of the similar anticoagulant compounds resulted from step 2.2, and their anticoagulant effect was not reported previously. According to these principles, 15 medicinal plants of Iran were selected to be evaluated for their anticoagulant effect. Then the selected anticoagulant plants were purchased from Omidvar, a specialized plant shop (Grand Bazaar, Tehran, Iran). Subsequently, the identity of the purchased plants was checked by an expert botanist, and a voucher specimen was appointed to each plant in the herbarium of Bioinformatics and Drug Design Unit, Pasteur Institute of Iran, Tehran (Table 1). Plants were chopped individually for the extraction procedure. Extraction was completed by the percolation technique in which 100 grams of plant powder were dissolved in 300 ml of 80% ethanol and retained in a sealed dish for 24 hours. This process was done three times at room temperature (25ºC) to extract all the active ingredients of the plants. Each plant extract was then condensed by an evaporator device in which, using a vacuum at temperatures below 50°C, all ethanol evaporated, and then the resulting extract was refrigerated at 4°C[13]. To provide a stock of extracts, 10 mg of the condensed extract of each plant was dissolved in one ml DMSO (Merck, Germany). Eventually, from each plant extract, three concentrations (1, 10, and 100 µg per ml) were prepared in DMSO for use in the blood coagulation test[14].

**Table 1:** The 15 selected medicinal plants with the help of cheminformatics approaches

No.	Voucher specimen	Plant	Used part of the plant	Place of cultivation	Major chemical compounds (%)
1	P148-16	*Dracocephalum moldavica*	Flower and leaves	Varamin, Iran	Acacetin (16), Diosmetin (10), salvigenin (6)[16]
2	P148-17	*Vachellia nilotica*	Seed	Isfahan, Iran	Rutin (28.5), Apigenin(10.5), Luteolin (9), Vanillic acid (0.36), Ellagic acid (0.57)[17]
3	P148-18	*Dianthus caryophyllus* L.	Dried bud	Mahalat, Iran	Hexadecanoic acid (28.7), Benzyl benzoate (12.6), Eugenol (18.22)[18]
4	P148-19	*Elettaria cardamomum* L.	Seed	India	Terpenyl acetate (64.5), Cineole (11.8), Terpineol (5.51), Limonene (2.23)[19]
5	P148-20	*Zea mays* L.	Seed	Ardabil, Iran	N/A
6	P148-21	*Marrubium vulgare* L.	Flower and leaves	Mazandaran, Iran	Eudesmol (11.93), Germacrene (9.37), Citronellyl formate (9.50), Citronellol (9.90)[20]
7	P148-22	*Rumex acetosella* L.	Flower and leaves	Isfahan, Iran	N/A
8	P148-23	*Terminalia bellirica* Roxb.	Fruit	Indonesia	Tannins (40%: Gallic acid, Sitosterol, Ellagic acid)[21]
9	P148-24	*Acacia senegal* L.	Resin	India	Arabinogalactan, diferulic acid[22]
10	P148-25	*Astragalus arbusculinus*	Resin	Shiraz, Iran	N/A
11	P148-26	*Centaurea depressa*	Flower	Ardabil, Iran	Piperitone (35.2), Elemol (14.1), β-Eudesmol (6.9), Spathulenol (5), Caryophyllene oxide (4.0), Hexadecanoic acid (4)[23]
12	P148-27	*Origanum vulgare* L.	Flower and leaves	Yazd, Iran	Caryophyllene (14.4), Spathulenol (11.6), Germacrene (8.1), α-Caryophyllene (2.7)[24]
13	P148-28	*Styrax officinalis* L.	Resin	Shiraz, Iran	2-Hexenal (20.7), Hexanol (5.8), Octanol (8.1), Geraniol (10.4)[25]
14	P148-29	*Juniperus sabina* L.	Seed	Isfahan, Iran	Sabinene (50.31), Elemol (5.74), Limonene (7.50), Terpinene (3.62), α-Pinene (7.97), β-Pinene (3.71), Terpinen-4-ol (3.79)[26]
15	P148-30	*Solanum melongena* L.	Seed	Tehran, Iran	Chlorogenic acid, Caffeic acid, Nasunin[27]

N/A, not available (according to literature review)

### *In vitro* coagulation test

The *in vitro* coagulation tests, prothrombin time (PT) and activated partial thromboplastin time (APTT), were carried out by a practiced lab technician. Initially, 5 ml of blood from seven normal candidates with identical blood group was collected and mixed together and then transferred to a tube containing sodium citrate 3.8%, as the anticoagulant. Next, the blood samples were centrifuged at 2016 ×g for 10 minutes to acquire required platelet-rich plasma (PRP). In PT test, 100 µl of PRP was mixed with 20 µl of each concentration of the plant extract and incubated at 37°C for 5 minutes. Lastly, 200 µl of PT assay reagent (Liquplastin, Bahar Afshan, Iran), preincubated at 37ºC, was added to the mixture, and the PT clotting time was recorded. For APTT test, 100 µl of PRP were mixed with 20 µl of each concentration of the plant extract and incubated at 37°C for 5 min. Then 100 µl of APTT assay reagent (Liqucephal, Bahar Afshan, Iran) was added and incubated for 1 minute. Finally, 100 µl CaCl_2_, preincubated at 37ºC, was added, and the APTT clotting time was recorded[15]. Furthermore, heparin (Alborz Darou, Iran; in three concentrations of 0.01, 0.1, and 1 IU) and *Glycyrrhiza glabra* L. in three concentrations (similar to plant extracts) were used as positive controls along with DMSO as the negative control.

### Statistical analysis

The resulting data are shown as the means ± standard deviation of three independent experiments. The statistical significance between control and treatment groups was determined by a paired student *t*-test and among multiple groups by one way analysis of variance (ANOVA), followed by Dunnett’s test. The significance level was set at *P*<0.05.

## RESULTS

### Search results for anticoagulant compounds and plants

According to search results, 27 anticoagulant compounds and 58 anticoagulant plants were identified, which are represented in our previous work[11].

### Results of cheminformatics approaches

In the results of similarity search, around 1,000 compounds similar to the anticoagulant compounds were found. Among the similar compounds, 15 medicinal plants of Iran, which comprised of the similar compounds and also their anticoagulant effect was not reported previously, were selected for coagulation test. These plants, their voucher specimen, place of cultivation, their major chemical compounds and the part used are listed in Table 1.

### Results of *in vitro* coagulation tests

**Fig. 1:**
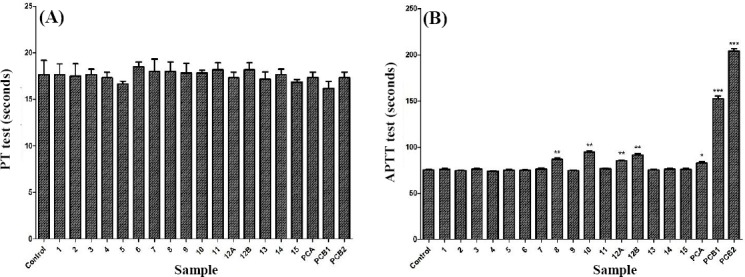
The effect of 15 selected medicinal plants on prothrombin time (A) and activated partial thromboplastin time (B) analyzed by one way analysis of variance (ANOVA) and Dunnett’s post-test. Samples: DMSO (Control), *Dracocephalum moldavica* (1), *Vachellia nilotica* (2), *Dianthus caryophyllus* (3), *Elettaria cardamomum* (4), *Zea mays* (5), *Marrubium vulgare (6), Rumex acetosella* (7), *Terminalia bellirica* (8), *Acacia senegal* (9), *Astragalus arbusculinus* (10), *Centaurea depressa* (11), *Origanum vulgare* (10 µg/ml; 12A), *Origanum vulgare* (100 µg/ml; 12B), *Styrax officinalis* (13), *Juniperus sabina* (14), *Solanum melongena* (15), *Glycyrrhiza glabra* (PCA) in concentration of 100 µg per ml, and heparin in concentrations of 0.01 IU (PCB1) and 0.1 IU (PCB2). *P<0.05, **P<0.01, and *** *P*<0.001 as compared to the control (standard *t*-test; means±standard deviation; n=3).

Based on coagulation test, three plants, including *Terminalia bellirica* (*P*=0.0019), *Astragalus arbusculinus* (*P*=0.0021), and *Origanum vulgare* (*P*=0.0014) with highest concentration (100 µg/ml) were found. Also, *Origanum vulgare* (*P*=0.0033) in concentration of 10 µg/ml presented a noteworthy anticoagulant effect on APTT test compared to negative control DMSO (Fig. 1). However, none of the plant extracts showed any effect on the PT test similar to our previous work[11]. In addition, two control positive samples, including *Glycyrrhiza glabra* (*P*=0.0142, 100 µg/ml) and heparin (*P*=0.0004, 0.01 IU and *P*=0.0002, 0.1 IU), showed a notable effect on APTT test compared to the negative control (Table 2, Fig. 1).

**Table 2:** Results of *in vitro* coagulation test

Sample^a^	Dose (µg/ml)	APTT (Seconds)	PT (Seconds)
Control	DMSO	75.30±0.57	17.66±1.53
*Dracocephalum moldavica*	100	76.00±1.00	17.66±1.15
*Vachellia nilotica*	100	74.60±0.57	17.50±1.32
*Dianthus caryophyllus*	100	76.16±0.76	17.60±0.57
*Elettaria cardamomum*	100	73.83±0.76	17.30±0.57
*Zea mays*	100	75.00±1.00	16.66±0.28
*Marrubium vulgare*	100	75.00±0.50	18.50±0.50
*Rumex acetosella*	100	76.16±1.04	18.00±1.32
*Terminalia bellirica*	100	86.83±1.25**	18.00±1.00
*Acacia Senegal*	100	74.60±0.57	17.83±1.04
*Astragalus arbusculinus*	100	94.60±1.52**	17.83±0.28
*Centaurea depressa*	100	76.50±0.50	18.16±0.76
*Origanum vulgare*	10	85.30±0.57**	17.33±0.57
*Origanum vulgare*	100	91.50±1.50**	18.16±0.76
*Styrax officinalis*	100	75.30±0.57	17.16±0.76
*Juniperus sabina*	100	76.16±0.76	17.66±0.57
*Solanum melongena*	100	75.83±1.04	16.83±0.28
*Glycyrrhiza glabra^b^*	100	82.66±1.53*	17.30±0.57
Heparin^b^	0.01 IU	152.66±2.51***	15.50±0.50
Heparin^b^	0.1 IU	204.00±2.64***	17.33±0.57
Heparin^b^	1 IU	NC^c^	NC^c^

a Each value represents the means±standard deviation (n=3).

b Positive control;

c No clotting;

* *P*<0.05,

** *P*<0.01, and

*** *P*<0.001 as compared to the control (standard *t*-test).

## DISCUSSION

In the present study, the main objective was to evaluate the efficacy of cheminformatics approach indrug design and to study the anticoagulant effect of selected candidate plants, as well as to anticipate the possible additive effect of medicinal plants in case of concomitant use of anticoagulant medication. Therefore, we successfully identified an anticoagulant influence in three out of 15 evaluated plants. Hence, following our previous work[11], we evaluated the anticoagulant effect of 30 different native medicinal plants of Iran, which eight of which showed a significant *in vitro* anticoagulant effect. According to our results, in the continuation of the previous study[11], we confirmed the usefulness and cost-effectiveness of the cheminformatics methods to predict and to discover potential new therapeutic compounds and plant materials. Also, we could effectively show the suitability of PT and APTT coagulation tests to evaluate the effect of plant extracts on the coagulation system *in vitro*.

The three medicinal plants with recognized anticoagulant effect, including *Terminalia bellirica*, *Astragalus arbusculinus*, and *Origanum vulgare*, are suitable candidates to be considered as candidate herbal medicines in the prevention and treatment of cardiovascular diseases, such as heart attacks and strokes. However, more evidence like *in vivo* study is required to understand the safety and the real effect of these plants. Regarding the effect of heparin, concomitant use of those plants with positive *in vitro* anticoagulant effect and anticoagulant drugs (e.g. heparin) will have an increased effect on coagulation[6]. Therefore, caution should be taken by patients who consume anticoagulant drugs and medicinal plants simultaneously, due to high risk of excessive bleeding and other unknown effects. Moreover, determining the active ingredients of the plants in order to produce plant-based anticoagulant compounds and medicines would be helpful.
